# *Spexwavepy*: an open-source Python package for X-ray wavefront sensing using speckle-based techniques

**DOI:** 10.1107/S1600577524005861

**Published:** 2024-07-30

**Authors:** Lingfei Hu, Hongchang Wang, Kawal Sawhney

**Affiliations:** a National Synchrotron Radiation Laboratory University of Science and Technology of China Hefei Anhui230029 People’s Republic of China; b Diamond Light Source Harwell Science and Innovation Campus DidcotOX11 0DE United Kingdom; IOM-CNR and Elettra-Sincrotrone, Italy

**Keywords:** *spexwavepy*, X-ray optics, wavefront sensing, speckle tracking, Python packages

## Abstract

*In situ* wavefront sensing plays a critical role in the delivery of high-quality beams for X-ray experiments. X-ray speckle-based techniques stand out among other *in situ* techniques for their easy experimental setup and various data acquisition modes. Although X-ray speckle-based techniques have been under development for more than a decade, there are still no user-friendly software packages for new researchers to begin with. Here, we present an open-source Python package, *spexwavepy*, for X-ray wavefront sensing using speckle-based techniques. This Python package covers a variety of X-ray speckle-based techniques, provides plenty of examples with real experimental data and offers detailed online documentation for users. We hope it can help new researchers learn and apply the speckle-based techniques for X-ray wavefront sensing to synchrotron radiation and X-ray free-electron laser beamlines.

## Abstract 2

An open-source Python package for X-ray wavefront sensing using speckle-based techniques, namely *spexwavepy*, has been presented.

## 1. Introduction

Modern synchrotron radiation facilities and X-ray free-electron lasers provide advanced platforms for multidisciplinary researchers around the world to benefit from high-brilliance X-rays. Cutting-edge scientific and industrial demands are driving the desire for continual improvements in X-ray quality. Along with the various endeavours undertaken to fulfil this desire, continuous progress has been made as well in assessing the quality of the wavefront during the transportation of the X-ray beam. As is often quoted as ground truth: ‘If you can’t measure it, you can’t improve it’. There have been many efforts to improve the accuracy and precision of both *ex situ* visible light metrology (Takacs & Qian, 1997 ▸; Yamauchi *et al.*, 2003 ▸; Siewert *et al.*, 2008 ▸, 2012 ▸; Alcock *et al.*, 2010, 2016 ▸ ▸; Assoufid *et al.*, 2013 ▸; Nicolas & Martínez, 2013 ▸; Idir *et al.*, 2014 ▸; Huang *et al.*, 2018 ▸; da Silva *et al.*, 2023 ▸) and *in situ* at-wavelength measurements (Hignette *et al.*, 1997 ▸; Yumoto *et al.*, 2006 ▸; Idir *et al.*, 2010 ▸; Kewish *et al.*, 2010 ▸; Wang *et al.*, 2011 ▸; Sutter *et al.*, 2012 ▸; Assoufid *et al.*, 2016 ▸; Laundy *et al.*, 2019 ▸; Moxham *et al.*, 2021 ▸).

When shining a laser through a diffuser, a 2D random intensity (speckle) pattern appears. The speckle-based techniques use these speckle patterns as probes to retrieve the wavefront information. These techniques have long been used in visible light metrology and other applications (Goodman, 2007 ▸, 2015 ▸). These techniques were extended to the X-ray region a decade ago by early researchers (Bérujon *et al.*, 2012 ▸; Morgan *et al.*, 2012 ▸). Since then, X-ray speckle-based techniques have burgeoned (Berujon *et al.*, 2014 ▸, 2020*a* ▸,*b* ▸; Wang, Kashyap *et al.*, 2015 ▸, Wang, Sutter *et al.*, 2015 ▸; Zhou *et al.*, 2018 ▸; Qiao *et al.*, 2020 ▸; Rebuffi *et al.*, 2023 ▸; Shi *et al.*, 2023 ▸). They stand out due to the relatively simple experimental setup and various data acquisition modes (Hu *et al.*, 2022*a* ▸,*b* ▸, 2023 ▸).

Although X-ray speckle-based techniques have great potential for X-ray wavefront sensing, there are only a few software packages (Berujon *et al.*, 2020*b* ▸; Vo *et al.*, 2021 ▸) currently available for data processing. They either lack well organized documentation and easy-to-use APIs (application programming interfaces) (Berujon *et al.*, 2020*b* ▸) or focus on X-ray imaging rather than the various modes of wavefront sensing techniques (Vo *et al.*, 2021 ▸). In this paper, we present an open-source Python package dedicated to the speckle-based wavefront sensing techniques for X-ray optics. The package is called *spexwavepy*, which is an abbreviation for the *spe*ckle-based *X*-ray *wave*front sensing *py*thon package. This package is designed to help new researchers learn and use the speckle-based techniques for X-ray wavefront sensing. It provides rich documentation to cover the various data acquisition modes and aims to teach new users about the data processing of speckle-based techniques from scratch. This Python package can assist users with the data processing of their wavefront sensing experiments.

## 2. The speckle-based wavefront sensing techniques

**Figure 1:**
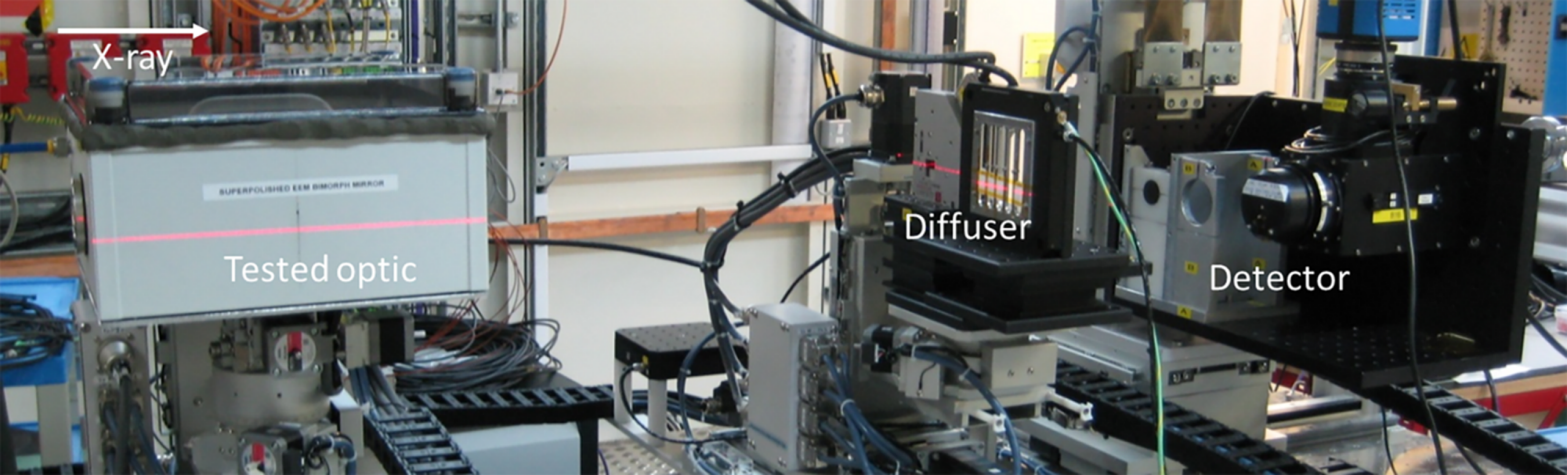
Typical setup for the speckle wavefront sensing experiment. This photograph was taken at the Test beamline B16 (Sawhney *et al.*, 2010 ▸) at Diamond Light Source.

A typical experimental setup for hard X-rays is shown in Fig. 1 ▸. For hard X-rays, one sheet or several stacked sheets of sandpaper can be used as the diffuser (Hu *et al.*, 2022*a* ▸). Recently, a coded mask to be used as a diffuser for X-ray wavefront sensing has been reported (Shi *et al.*, 2022 ▸). The diffuser is often mounted on a 2D scannable high-precision stage. Usually, a scintillator is used to convert the incident X-rays to visible light for detection. The whole detecting system is also coupled with a visible-light microscope to improve spatial resolution.

In general, the speckle-based X-ray wavefront sensing methods can be divided into two modes. They depend on whether the reference beam is available or not. If the tested optic focuses the beam weakly, such as a single CRL (compound refractive lens) or even a planar reflecting mirror, the incident beam without the tested optic is used as a reference beam. The images with speckle patterns acquired with and without the tested optic are compared. The data acquisition modes for this type of method are called ‘differential modes’ in other literature (Berujon *et al.*, 2020*a* ▸). In this package, these data acquisition modes are labelled with the suffix ‘with reference beam’. For a strongly focusing optic such as a curved mirror, the speckle patterns can be very different with and without the tested optic in the beam. In this case, no reference beam is available. The images whose speckle patterns are compared are all taken with the tested optic in the beam. In this package, these data acquisition modes are labelled with the prefix ‘self-reference’. These modes are called ‘absolute modes’ in other literature (Berujon *et al.*, 2020*a* ▸).

All data acquisition modes trace the shift of a speckle pattern. However, the shift can represent different physical quantities for different experimental methods. In brief, when a reference beam is available, the speckle pattern shift is caused by the first derivative, *i.e.* the slope, of the measured wavefront. Otherwise, in the self-reference mode, the speckle pattern shift is caused by the second derivative, *i.e.* the curvature, of the measured wavefront. Another variation of the experimental method is the position of the diffuser. It can be placed upstream or downstream of the tested optic, depending on the type of optic. In general, if there is a reference beam, as in the case of a weakly focusing optic, the shift of the speckle pattern from the reference image to the compared image is due to the local wavefront slope change in the plane of the diffuser for the downstream case and in the optical central position for the upstream case. On the other hand, in the self-reference mode, the shift of the speckle pattern can be attributed to the local wavefront curvature change. Similarly, the curvature change is measured at the position of the diffuser for the downstream case but in the optical central position for the upstream case. Apart from that, the algorithms used to reconstruct the local wavefront curvature for the two cases are also different (Berujon & Ziegler, 2012 ▸; Zhou *et al.*, 2024 ▸). This is in contrast to the case with a reference beam.

Table 1 ▸ provides a summary of the speckle-based techniques included in this package. We assume the incident beam is a quasi-parallel beam from the synchrotron radiation source going through the beamline without any other optics except one monochromator. If the incident beam is a quasi-spherical wave, some modifications are needed for certain techniques when processing the data. Adaptations to quasi-spherical incident wavefronts are left to the discretion of the users. The speckle-based techniques are very flexible and there are some other experimental methods. For instance, due to special experimental considerations, all the techniques included in *spexwavepy* keep the detector fixed. As a result, none of the so-called ‘absolute mode’ speckle-based techniques (Berujon *et al.*, 2020*a* ▸,*b* ▸) are included in this Python package explicitly. However, we ask the users to compare the ‘new’ data acquisition mode with the existing ones before requiring the developers to implement it. For example, the ‘absolute mode’ XST technique (Berujon *et al.*, 2020*a* ▸,*b* ▸) is equivalent to the conventional XST with a reference beam in this package, only replacing the distance between the diffuser and the detector plane with the distance of the movable detector. Comparison with the existing data acquisition methods in this package requires the users to understand the underlying physics of each experiment. These methods have been introduced in the documentation provided with this package.

## 3. Package structure and features

### 3.1. Modules of *spexwavepy*

**Figure 2:**
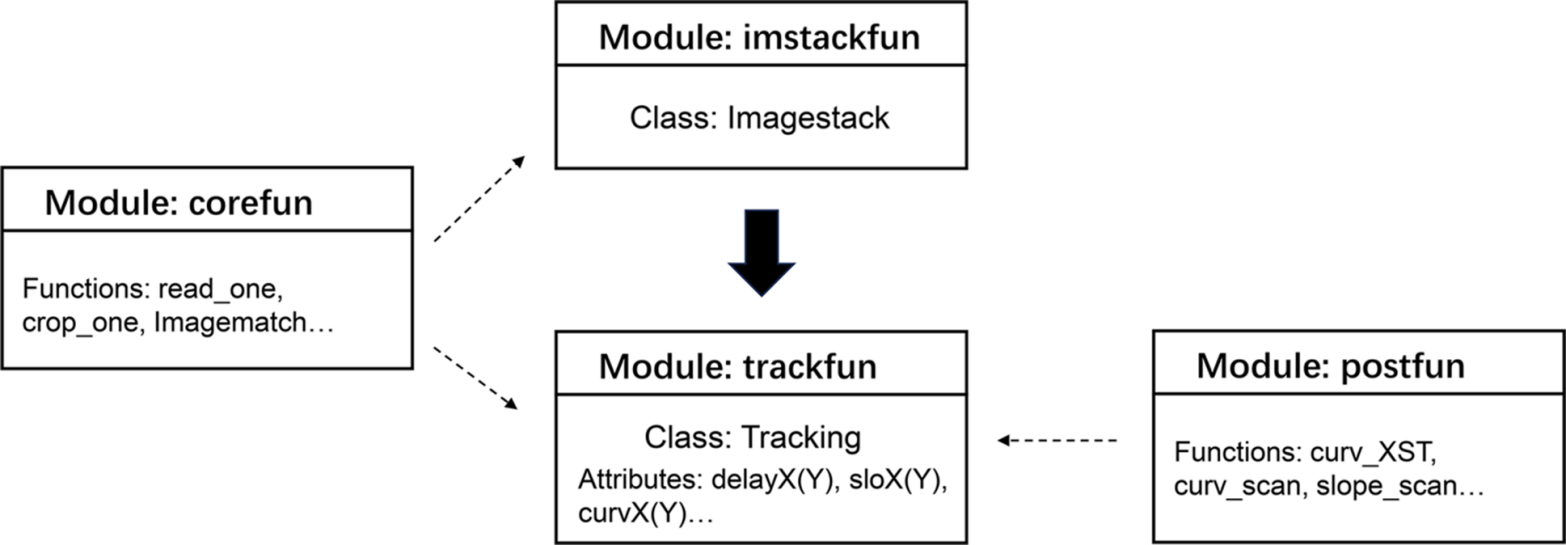
Modules of *spexwavepy*. The *Imagestack* and *Tracking* classes should be defined in sequence.

Two Python classes, *Imagestack* and *Tracking*, need to be defined in order to use *spexwavepy*. The two classes are included in the *imstackfun* and *trackfun* modules, respectively. Fig. 2 ▸ summarizes the four modules that make up this Python package. The *Imagestack* class is the container for the raw data and is the first class that is needed for data processing. Its methods preprocess the raw images before the critical shift tracking of the speckle pattern. This class is then used as input for the *Tracking* class. The attributes of the *Tracking* class, such as *delayX*, *delayY*, *sloX*, *sloY*, *curvX*, *curvYetc.* store the reconstructed physical quantities according to the type of data acquisition mode. The *corefun* and *postfun* modules provide the auxiliary functions.

### 3.2. Features of *spexwavepy*

#### 3.2.1. Data acquisition modes

*Spexwavepy* includes most reported X-ray speckle-based techniques (see Table 1 ▸). Some techniques in the literature may not be explicitly provided in this package. However, some of these are included in *spexwavepy* under other names just because of the ambiguity in the naming of the techniques, while others do not require a new method to be defined because they deviate only in minor ways from data acquisition modes already in *spexwavepy*. Even if some novel modes are missing, they are relatively easy to implement by the authors or even the users since the code of this package is modularized and open-source.

#### 3.2.2. Parallelized data processing procedure

The 2D speckle tracking can sometimes be time-consuming. We used the built-in *multiprocessing* module of Python to parallelize the code to speed up the data processing procedure. The multiprocessing module supports the process-based parallelism. The code can distribute the 2D speckle tracking among the multiple CPUs provided by modern computers. The user needs to define the available number of CPUs in advance. All the multi-core versions of the tracking modes end with the ‘multi’ suffix. The code was designed for Linux, but it may fail in Windows because the multiprocessing modules in these two operating systems fork a new process from its parent in different ways. We are working to extend the usage of parallel computing for the Windows user of this package at present.

#### 3.2.3. Examples and documentation

**Figure 3:**
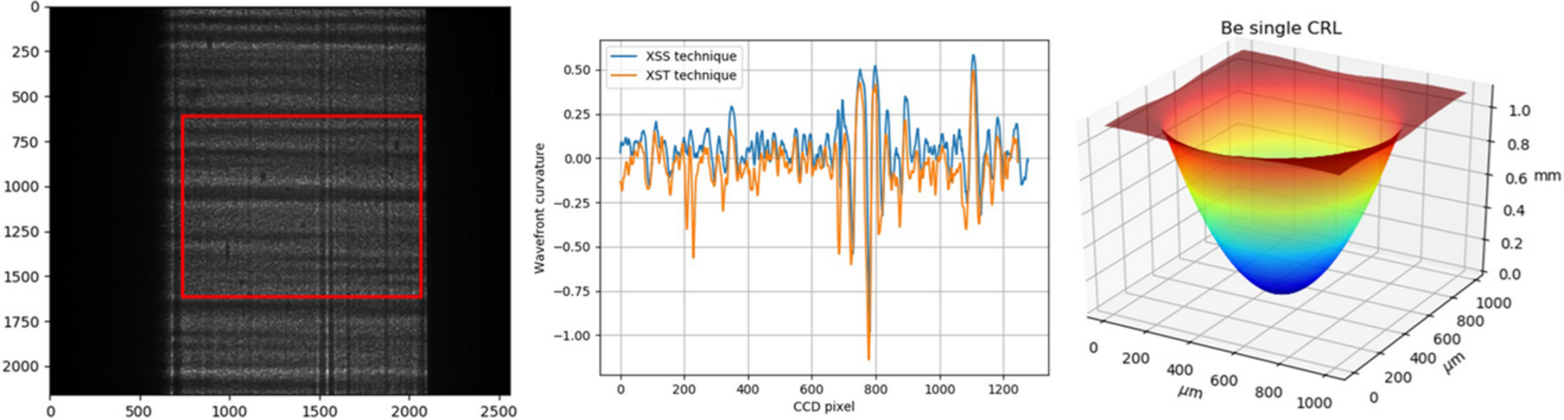
Banner of the online documentation. Left to right: the raw speckle image after a tested plane mirror, comparison between the self-reference XSS technique and the self-reference XST technique, reconstructed profile of a single CRL.

We developed this Python package to help new researchers learn the speckle-based X-ray wavefront sensing techniques. We hope this package can help researchers to process their data. Although there are some packages for the data processing of speckle-based X-ray wavefront sensing (Berujon *et al.*, 2020*a* ▸,*b* ▸), they are far from user-friendly, and they lack detailed documentation and helpful examples. We reckon that being user-friendly is paramount for a package to be used widely. To do that, we provide rich documentation and examples to help users become familiar with this package. Almost all the examples included in the package are excerpted from our previously published paper (Hu *et al.*, 2021 ▸, 2022*a* ▸,*b* ▸, 2023 ▸; Zhou *et al.*, 2024 ▸). In addition, we also shared the experimental data. The users can reproduce the results from the shared data. The online documentation of *spexwavepy* can be found at https://spexwavepy.readthedocs.io/en/latest/. Fig. 3 ▸ shows the banner of the online documentation.

## 4. Computational consumption

*Spexwavepy* has been developed and tested mainly on two Linux workstations. Table 2 ▸ provides the basic information of the workstations. The 2D speckle tracking can take quite a long time depending on the size of the raw images. It can take more than 1 h if the images are large enough and the code runs on a single CPU. The speckle tracking methods can run on multiple CPUs owing to the Python built-in *multiprocessing* package. We have developed the multiprocessing version of each function. The multiprocessing package can help to reduce the processing from around 1 h on one CPU to less than 20 min on 16 CPUs. More CPUs will further reduce the processing time.

## 5. Example: measurement of the wavefront local curvature after a plane mirror

**Figure 4:**
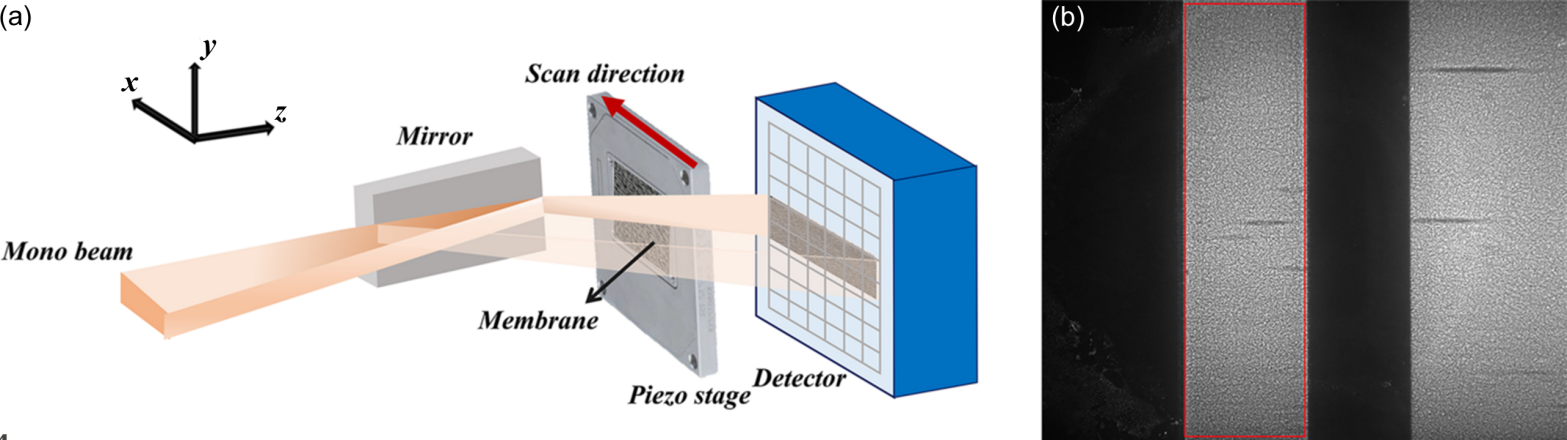
(*a*) Experiment setup of the self-reference XSS technique. The diffuser scans in the *x* direction, which is along the mirror length. (*b*) One raw image in the image stack. The area within the red box is the reflected beam from the tested plane mirror. The other bright area is the direct beam from the source.

We use an example code to demonstrate the usage of the *spexwavepy* package. We measure the wavefront local curvature after a plane mirror in this example. We use the self-reference X-ray speckle scanning (XSS) technique to obtain the wavefront local curvature. Fig. 4 ▸(*a*) shows the experimental setup for this technique. The diffuser is downstream of the tested plane mirror. It scans in the *x* direction for a better spatial resolution along the mirror length. Thus, there will be a series of images. Fig. 4 ▸(*b*) shows one raw image in a stack of 301 images.

The physical quantity directly derived from the self-reference XSS technique by tracking the shift of the speckle pattern is the wavefront curvature, *i.e.* the second derivative of the wavefront (Wang, Sutter *et al.*, 2015 ▸). In this example, the diffuser scans along the *x* direction. We can obtain the wavefront local curvature 1/*R_x_* in this direction:
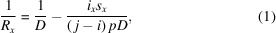
where *i_x_* is the tracked shift of the speckle pattern in the *x* direction, *s_x_* is the scan step size, *i* and *j* are the column numbers of the image when the tested mirror is placed vertically [as in Fig. 4 ▸(*a*)], *j* − *i* is usually a fixed value equal to 2 or 3, *p* is the pixel size of the detector, and *D* is the distance between the diffuser and the detector plane in this case.

**Figure 5:**
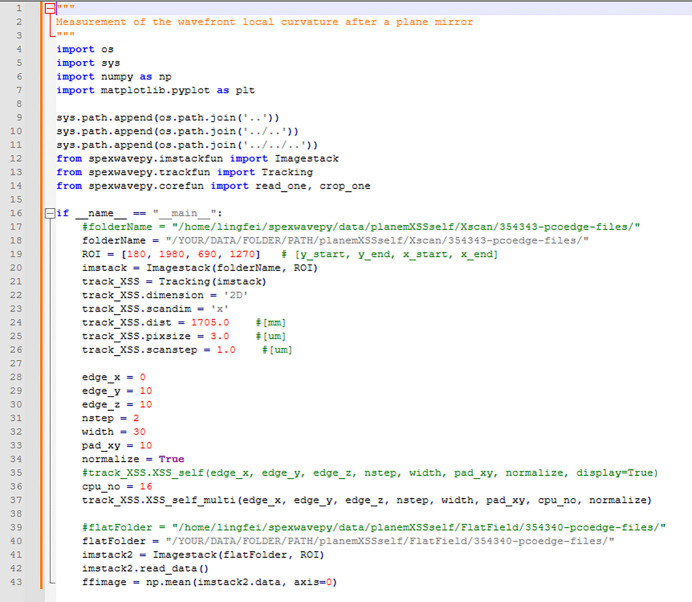
Sample code, *plane_XSSself.py*, in the examples folder.

See Fig. 5 ▸ for a sample code. This example is also in the released package (*plane_XSSself.py* in the examples folder). The image stack is loaded into the *Imagestack* class. The ROI is set to extract the reflected beam. Then, the *Imagestack* class is the input of the *Tracking* class. The scan direction, scan step size, distance between the diffuser and the detector, pixel size *etc.* are also defined in the *Tracking* class. After defining some necessary parameters for calculating the local wavefront curvature, we call the *XSS_self* method of the *Tracking* class or its multiprocessing form *XSS_self_multi* to do the speckle tracking. The 2D map of the wavefront local curvature in the *x* direction is stored in the *curvX* attribute of the *Tracking* class.

**Figure 6:**
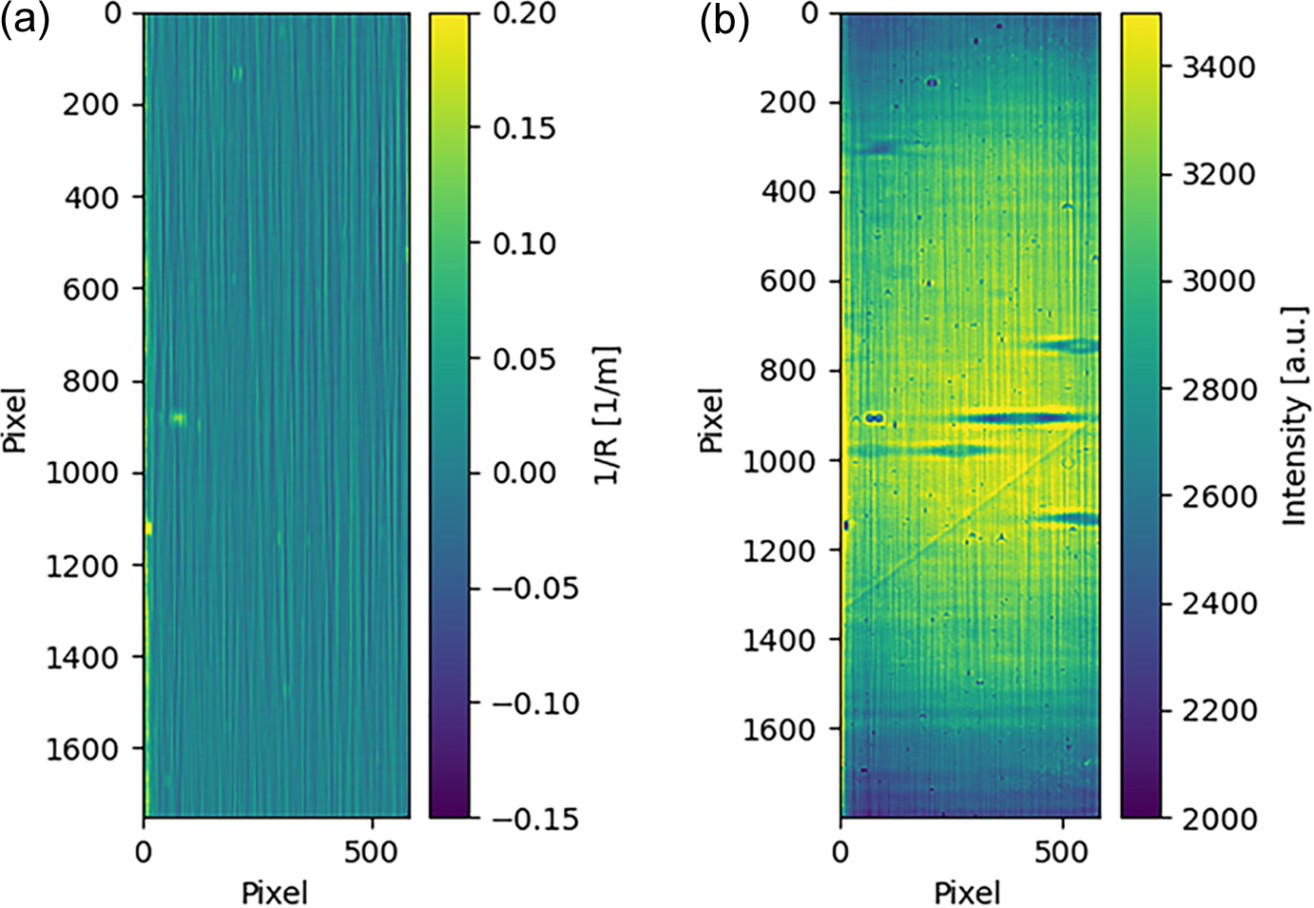
(*a*) Wavefront local curvature distribution obtained. (*b*) Far-field intensity distribution on the detector.

Fig. 6 ▸(*a*) shows the wavefront local curvature obtained. Note the resemblance of its vertical stripes to those in Fig. 6 ▸(*b*). Fig. 6 ▸(*b*) is the intensity distribution after the tested plane mirror. This phenomenon is not a coincidence. We can set the relationship between the far-field intensity distribution and the wavefront local curvature; however, this topic is beyond the scope of this paper. Please refer to the related works (Hu *et al.*, 2021 ▸, 2023 ▸).

## 6. Conclusions

We have presented an open-source and modularized Python package, named *spexwavepy*, for data processing of speckle-based X-ray wavefront sensing techniques. We hope it can help new researchers to learn and apply speckle-based techniques for X-ray wavefront sensing to synchrotron radiation and X-ray free-electron laser beamlines. For that purpose, we not only share the source code but also provide detailed documentation introducing the various speckle-based techniques and the implementation of the code. We also created several examples with shared experimental data to help users become familiar with this package. *Spexwavepy* includes several proposed data acquisition modes. We believe this package can deal with various experimental conditions. Even if some experimental modes are missing, they are easy to add to the current package. The documentation and the shared experimental data are both online. At present, we use the built-in Python multiprocessing package for parallel computing. It limits the code to run on a single machine. Some other parallelization schemes, such as MPI, are under consideration.

## Figures and Tables

**Table 1:** Summary of the speckle-based techniques included in *spexwavepy* XST = X-ray speckle tracking. XSS = X-ray speckle scanning. XSVT = X-ray speckle-vector tracking.

Technique	Number of images	Physical quantity directly measured
Conventional XST	One image for both the reference and the sample datasets.	Wavefront slope
Self-reference XST	Two images for the sample dataset.	Wavefront curvature
XSS with reference beam	The number of images in both the reference and the sample datasets is the same and is equal to the number of scans.	Wavefront slope
Self-reference XSS	Sample dataset only. The number of images is equal to the number of scans.	Wavefront curvature
XSVT	The number of images in both the reference and the sample datasets is the same and is equal to the number of random scans.	Wavefront slope

**Table 2:** Two workstations used to develop and test *spexwavepy*

Workstation	Operating system	Memory	Number of CPUs
Intel(R) Xeon(R) CPU E5-2680 v3 at 2.50 GHz	Red Hat Enterprise Linux Workstation 7.9 (Maipo)	125 G	24
Intel(R) Xeon(R) CPU E5-2670 v3 at 2.30 GHz	Rocky Linux 8.7	125 G	48

## Data Availability

The source code and the latest developments of *spexwavepy* are available at GitHub (https://github.com/wholingfei/spexwavepy/) and the Python Package Index (PyPI) repository (https://pypi.org/project/spexwavepy/). It can be easily installed using Python package installer (*pip*). The data used in the examples are available at https://zenodo.org/records/10892838. The documentation of *spexwavepy* can be found at https://spexwavepy.readthedocs.io/en/latest/.
